# Filling the gap: Micro-C accesses the nucleosomal fiber at 100–1000 bp resolution

**DOI:** 10.1186/s13059-015-0744-8

**Published:** 2015-08-21

**Authors:** Julien Mozziconacci, Romain Koszul

**Affiliations:** Theoretical Physics for Condensed Matter Laboratory, UPMC, CNRS UMR 7600, Sorbonne Universités, Place Jussieu, 75005 Paris, France; Institut Pasteur, Department Genomes and Genetics, Groupe Régulation Spatiale des Génomes, 75015 Paris, France; CNRS, UMR 3525, 75015 Paris, France

## Abstract

The fine three-dimensional structure of the nucleosomal fiber has remained elusive to genome-wide chromosome conformation capture (3C) approaches. A new study mapping contacts at the single nucleosome level (Micro-C) reveals topological interacting domains along budding yeast chromosomes. These domains encompass one to five consecutive genes and are delimited by highly active promoters.

## Introduction

Over the past ten years, genome-wide derivatives of the chromosome conformation capture approach (3C [1] and Hi-C [2]) have provided important mechanical and functional insights into the organizational principles of eukaryotic and prokaryotic genomes. A broad range of intra-chromosomal structures have been described, including gene loops [3], chromosome domains that are enriched in self-contacts, and large regulatory loops [2]. However, the experimental constraints of the 3C/Hi-C approach impose a limit to its resolution: the distribution of restriction sites along the chromosome is not uniform but rather follows a Poisson distribution, which is highly skewed by the local GC content and the presence of repetitive sequences. It is less likely that smaller restriction fragments will be cross-linked and trapped during the experiment than larger ones [4], so even frequent cutting enzymes (producing 4-bp fragments) fail to provide a resolution below 1 kb over the whole genome. The fine structure of the nucleosomal fiber in vivo has therefore remained out of the reach of these techniques. To investigate this blind spot, Hsieh and colleagues [5] designed and applied a new genomic approach, a micrococcal nuclease (Mnase) chromosome conformation assay dubbed Micro-C, in yeast *Saccharomyces cerevisiae*.

## Micro-C reveals multi-gene domains in *S. cerevisiae*

**Fig. 1:**
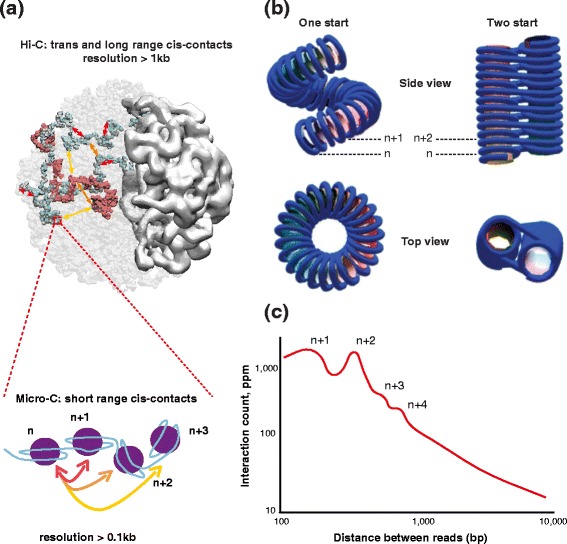
**a** Schematic representation of the differences between Micro-C and Hi-C contacts. *Top*: snapshot from a physical simulation of the yeast chromosomes as a brush of polymers (*white beads*) tethered by their centromeres (*yellow spheres*). The large structure on the right represents the nucleolus (see also [6]). Chromosomes 3 and 11 are highlighted in *red* and *cyan*. Each bead represents three nucleosomes. *Bottom*: expanded view of four nucleosomes (*purple circles*). Contacts, from frequent to rare, are highlighted on both representations in *red*, *orange* and *yellow*. **b** Models of the chromosomal fiber with 167-bp nucleosomal repeat length (built using the three angle model described in Riposo and Mozziconacci [9]). **c** Number of contacts in parts per million (ppm) obtained through a Micro-C experiment at various genomic distances (schematic representation of the results presented in Figure S3 of Hsieh et al. [5]). The first bump corresponds to contacts between nucleosome n and n + 1, and the second bump to contacts between n and n + 2. Only reads in the same orientation were used to avoid self-ligation artifacts

The Micro-C approach developed by Rando and colleagues [5] elegantly alleviates some of the 3C limitations by exploiting the regular spacing of nucleosomes (167 bp in yeast) along the DNA strand. After a formaldehyde fixation step the approach uses Mnase, instead of a restriction enzyme as in conventional 3C, to digest DNA. Mnase digests the accessible linker DNA between nucleosomes, providing access to the budding yeast chromatin fiber at a new level of resolution (Fig. 1a). Until now, *S. cerevisiae* chromosomes have essentially been described at larger scales as a dynamic brush of polymers tethered at their centromeres [6]. The analysis by Hsieh et al. of high-resolution contacts unveiled structural units that had escaped investigations using 3C-based approaches [7]. The Micro-C and classical 3C-based approaches are complementary as the Micro-C signal picks up considerably fewer long-range or inter-chromosomal interactions than does traditional 3C, as illustrated by the lack of centromere–centromere contacts. Interestingly, the gene-based structural units revealed by Micro-C are strongly reminiscent of the chromosome interaction domains (CID) identified in the bacteria *Caulobacter crescentus* [8], with the borders of the domains corresponding to the promoters of highly expressed genes. Hsieh et al. [5] also drew an interesting parallel between yeast CIDs and mammalian topologically associating domains (TADs) based on the average number of genes per domain (one to five). This parallel suggests that the formation of boundaries through the recruitment of regulatory and structural proteins is the key determinant of chromosome organization in eukaryotes.

## Implications for nucleosomal fiber models

In addition to investigating new links between fine chromosomal structures and transcription, the Micro-C assay gave the authors the opportunity to assess existing models of the yeast nucleosomal fiber. On the basis of the relatively short linker length between consecutive nucleosomes in yeast (20 bp) two alternative structures for the nucleosomal fiber have been proposed (see, for example, [9] for a review; Fig. 1b). Consecutive nucleosomes (n and n + 1) can be stacked upon each other, resulting in a columnar arrangement that has been proposed to be further wrapped into a solenoidal structure [10]. Alternatively, the closest neighbors in space can also correspond to nucleosomes that occupy every two positions linearly (n and n + 2), resulting in a zig-zag motif that can be further stabilized by nucleosome-stacking interactions. Surprisingly, the inter-nucleosomal contacts reported by Hsieh et al. are compatible with both models as the number of (n/n + 1) contacts is roughly similar to the number of (n/n + 2) contacts (schematized in Fig. 1c, but see Figure S3 of Hsieh *et al*. [5]). These findings suggest either a dynamic equilibrium between these two structures or the absence of a highly structured nucleosomal fiber. The lack of any periodicity at 4–6 nucleosome spacing strongly suggests that the columnar phase, if it exists, is not wrapped into any higher-order periodical structure, as was proposed in pioneering studies on the chromatin fiber structure [10]. In addition, it could be argued that the asynchronous populations used to perform the experiment contain diverse structures that correlate with the diverse stages of the cell cycle. Therefore, it may be interesting to perform Micro-C on synchronized cells to search for such effects.

In line with the possibility of a polymorphic structure, Hsieh et al. show that several factors can change what they describe as the ‘compaction’ of the chromosomal fiber. Here, the compaction is simply defined as the ratio of long-range over short-range contacts (with short-range being defined as closer than 300 bp). The compaction of each gene was found to be correlated with its transcriptional activity, and the decrease in compaction observed for highly transcribed genes might be attributed to the local disruption of the nucleosomal fiber by active RNA polymerase(s). Consistent with this finding, genes that were upregulated following a diamide treatment were convincingly shown to be less compacted.

## The players at work in shaping the nucleosomal fiber

To investigate the mechanistic basis of gene compaction further, the authors set out to repeat the Micro-C experiment in 23 *S. cerevisiae* mutants in which chromatin structure is altered. Micro-C confirmed the role of the RSC chromatin remodeling complex and the cohesin complex in chromatin structuring, with defects in these complexes being associated with increased gene compaction. Conversely, other chromatin mutants, such as those with defects in Mediator or the histone deacetylase Rpd3, appeared to induce a significantly reduced level of gene compaction. This change is, however, accompanied by only small changes in transcriptional activity. The effect of these ‘structural mutants’ suggests that changes in compaction can also be modulated by factors other than the progression of DNA polymerase along the genes, either by the potential formation of transient long-range loops or by the modification of inter-nucleosomal interactions. To test for the latter specifically, the authors used variants of histone H4 that were previously characterized in vitro as having a direct effect on nucleosome array folding. Micro-C confirmed the important role of the H4 N-terminal tail in vivo.

Finally, the authors took advantage of their high-resolution assay to search for the promoter and terminator gene loops identified previously using a 3C approach [3]. Quite unexpectedly, such structures were not apparent in their data. However, the transcription regulator protein Ssu72, reported to be essential for the loop formation, resulted in a small but significant reduction in gene compaction. These results suggest that the reports of loops may need to be looked at again through more globular or chromatin-accessibility focused lenses.

## Conclusions

Micro-C provides an additional tool to investigate the structure of chromatin at fine resolution. In the pioneering study by Hsieh and colleagues, the authors describe gene domains in *S. cerevisiae* at an unprecedented level of resolution, refining our understanding of chromosome organization in this species. Obviously, larger genomes can be investigated next and, if successful, this work will provide answers to hotly debated questions in the field.

## Abbreviations

3C: Chromosome conformation capture
CID: Chromosomes interaction domains
Mnase: Micrococcal nuclease
